# Protect, promote and support: a warm chain of breastfeeding for oncological women—results from a survey of young Italian cancer mothers

**DOI:** 10.3332/ecancer.2020.1151

**Published:** 2020-12-07

**Authors:** Martina Smorti, Ilaria Testa, Marta Gallese, Arianna Dotti, Chiara Ionio, Angelica Andreol, Anna Zilioli, Gabriella Pravettoni, Andrea Greco, Valentina Fenaroli, Giuseppe Nastasi, Nicola Giuntini, Lucia Bonassi

**Affiliations:** 1 Department of Surgical, Medical and Molecular Pathology and Critical Care Medicine, University of Pisa, Via Paradisa 2, 56126 Pisa, Italy; 2 ASST Bergamo Est Mental Health Department, via Paderno 21, 24068 Seriate BG, Italy; 3 Department of Psychology, Catholic University of Milan, L.go Gemelli 1, 20123 Milano, Italy; 4 ASST Bergamo Est Gynaecology-Obstetrical Department, via Paderno 21, 24068 Seriate BG, Italy; 5 Istituto Europeo di Oncologia Milano, Applied Research Division for Cognitive and Psychological Science, Viale Ripamonti 435, 20141 Milano, Italy; 6 Department of Human and Social Sciences, University of Bergamo, P.le Sant’Agostino 2, 24129 Bergamo, Italy; 7 ASST Bergamo Est Medical Department, via Paderno 21, 24068 Seriate BG, Italy; a https://orcid.org/0000-0002-5867-9859; b https://orcid.org/0000-0002-0163-3841; c https://orcid.org/0000-0002-9135-2938; d https://orcid.org/0000-0002-8086-2801; e https://orcid.org/0000-0002-5827-8369; f https://orcid.org/0000-0001-5353-4899

**Keywords:** breastfeeding, cancer, mental health, warm chain

## Abstract

The topic of lactation following cancer diagnosis will become increasingly more current. Although oncological research confirms that breastfeeding after cancer might be possible, there is a lack of guidelines and a good recommendation for oncological women. In the absence of specific recommendations, women with past cancer may be at higher risk for psychological distress related to breastfeeding.

The objective of this article was to analyse the experience of breastfeeding in new mothers with a history of cancer compared to women without a cancer diagnosis. First, we explored the impact of the cancer diagnosis on the breastfeeding choice. Second, we evaluated the relationship between different feeding methods and the mother’s mood states in women with and without a history of cancer.

The sample was composed of 74 mothers divided into two groups: 34 with a cancer history (clinical sample) and 40 without a cancer diagnosis (control group). Participants were requested to complete a questionnaire three months after childbirth which assessed: socio-demographic and clinical data, feeding modes (breastfeeding, formula and mixed feeding) and the profile of mood states (POMS).

Results showed that women in the clinical group breastfeed significantly less and use formula more than those in the control group. Moreover, in the clinical group, women who breastfeed feel reported higher levels of confusion (according to POMS) than mothers who bottle-feed or use a mixed feeding method. On the contrary, in the control sample, women who breastfeed feel significantly more vigorous than *puerperae* who bottle-feed or use mixed methods according to POMS.

Our findings suggest the need for a specific *warm chain* of support and the development of guidelines with clear and specific information for women with a cancer diagnosis in order to reduce their confusion around breastfeeding.

## Introduction

Breastfeeding has always prompted great interest and debate in the scientific literature. The work of Kaiser [1] represents a milestone in this subject. The authors urge healthcare workers to establish the (so-called) *warm chain* of support to create a skilled care network to assist mothers in building up confidence with breastfeeding, to train them appropriately and to prevent harmful practice.

There is extensive evidence in the scientific literature emphasising the positive effects of breastfeeding for both mother and child, namely, nutrients and antibodies in breast milk which can empower newborns’ immunocompetence [2]. On the other hand, breastfeeding has been proved to reduce the risk of developing breast cancer by 4%–5% per year of lactation [3]; such protective effect is amplified by the duration and exclusivity of breastfeeding [4].

For these reasons, women are often encouraged to breastfeed since the beginning of pregnancy and according to the recommendations of the World Health Organization [5]. However, not all women adhere to this advice after the birth of the baby, due to health and medical issues or for conflicting schedules [6].

Our work aims to analyse the experience and practice of breastfeeding in mothers with a history of cancer compared to women without an oncological diagnosis.

Even considering only the Italian data from last year, more than 20.000 women below the age of 50 years were diagnosed with an oncological disease with breast cancer (BC) being the most common malignant tumour in females in the fertile period [7].

Most women with breast cancer undergo breast-conserving surgery as part of their oncological surgical management, whereas others are selected for total mastectomy. Depending on the type and the site of surgery, there are potentially significant implications for breastfeeding. The success of the lactation from the treated breast is affected by the proximity of the incision to the nipple or areolar complex, the location of the tumour, the dose and the type of radiotherapy [8]. Nevertheless, women undergoing breast surgery for cancer should be reassured about the adequacy of milk production by a single breast and encouraged to seek early advice if lactating problems occur [8].

Recent oncological researches confirm that breastfeeding after cancer treatment is still viable [9]. Anyway, studies so far have been focused almost exclusively on breast cancer and on the impact of different oncologic treatments on breastfeeding [3, 10]. Currently, there are few studies addressing the psychological impact of breastfeeding in mothers with any cancer diagnosis along with breast tumour. Despite the lack of data, this subject is recently gaining great relevance and challenging everyday practice for clinicians. As a matter of fact, in contemporary Western societies, figures show a dramatic surge of new cancer diagnoses in the pre-menopausal age—taking into account this evidence along with the well-known trend of delaying pregnancy after the age of 30, the topic of breastfeeding after a history of cancer will involve an increasing number of women [8]. Moreover, assuming that lactation might postpone the oncological follow-up schedule and that there are no consensus guidelines or strong recommendations [11], it is likely that many women with previous cancer could take on breastfeeding with a great degree of uncertainty. In this respect, as previously noted, there is not yet an established ‘best-practice’ as the small number of existing studies in the literature is focused exclusively on women with a previous breast-cancer diagnosis, not taking into consideration women with other types of tumour [11]. To the best of authors’ knowledge, there is only one study so far to analyse the psychological impact of breastfeeding after cancer, regardless of the disease type [11]. The results of this investigation show a staggering confusion and deep sense of frustration from mothers in regard to expectations associated with lactation [11].

Namely, the lack of clear guidelines and recommendations on breastfeeding for women with cancer is conflicting with ongoing promotion of breastfeeding in the general population, enhanced by the perceived message that it is a matter of ‘moral choice’ associated with ‘optimal parenting’ as opposed to potential risk in choosing formula [12, 13]. As a result, women with cancer experience who do not breastfeed may be exposed to unjustified stress, frustration and guilt, which increase fatigue during the postpartum period. For this reason, assessing the feeding choice and the psychological experience related to it in women with previous cancer is of paramount importance for health care workers in order to help women to choose responsibly and support their decision. Medical staff should assess the specificity of each case and provide every patient with information, assistance and encouragement about the possibility and duration of breastfeeding, evaluating the psychological burden [14].

Moving from previous considerations, the primary objective of our study is to explore the impact of the cancer diagnosis on the breastfeeding choice. We aim to analyse whether women with previous oncologic history choose to breastfeed and for how long, compared to healthy mothers during the first 3 months post-partum. Since UNICEF Breastfeeding promotion and support guidelines recommend exclusive lactation until 6 months of the baby’s birth and complementary food with continued breastfeeding up to 2 years for all puerperal women [15], we anticipate a greater percentage of healthy women choosing to breastfeed when compared to cancer patients.

The second aim is to evaluate the relationship between different feeding methods and mother’s mood states in the group of women with cancer history versus healthy women. Our hypothesis is that healthy women who breastfeed show a better emotional adjustment compared to the cancer study group.

## Methods

### Participants and procedure

This study is a part of a larger study assessing the psychological impact of cancer history on the psychological well-being of women during pregnancy [16] and the post-partum period. According to the study design and the aims of the research, a consecutive sample of Italian new-mothers is being prospectively recruited from January 2017 to September 2019 at the three participating institutions: the Territorial Social and Health Authority (ASST) Bergamo Est, the Scientific Institutes of Hospitalization and Care (IRRCS) European Institute of Oncology, and the IRRCS San Matteo of Pavia. Pregnant women were invited to participate in the study in the waiting room of the Hospital Obstetric Unit before routine third trimester medical visit. Clinical group inclusion criteria were: a) age > 18 years; b) able to speak and read Italian; c) previous cancer disease. Control group inclusion criteria were a) age > 18 years old; b) able to speak and read Italian and c) physiologic disease. Women with an established psychiatric diagnosis, with serious medical complications from pregnancy, or having a baby with severe genetic conditions or congenital abnormalities were excluded from the study. The inclusion and exclusion criteria were defined and agreed with clinicians and participation in the study has been communicated and agreed with the care team of the patients. A psychologist in charge of the study at each centre has been responsible for the informed consent process with women of both groups, highlighting the aims and study protocol, whose participation included also an email survey questionnaire to be completed 3 months after childbirth. Anonymity was guaranteed, and participation was voluntary, with no monetary incentive, with the possibility of withdrawal from the study at any moment.

Women who met the inclusion criteria in the clinical group and in the control sample were 34 and 125, respectively. All eligible women were invited to take part in the study and agreed to participate. Thirty-four women in the study group and 125 controls meeting the inclusion criteria were consecutively enrolled in the given study period. Considering the sample-size discrepancy and for the sake of statistical analysis and study comparison, women from the control group were further selected according to obstetric characteristic to comparably match the study population. The final series of this study consists of 74 pregnant women divided into two groups: 1) clinical group: 34 pregnant women aged from 31 to 46 (*M* = 38.61 SD = 3.64) with history of cancer; 2) control sample: 40 pregnant women aged from 22 to 47 (*M* = 32.11; SD = 5.38) without cancer history.

### Measures

In agreement with the proposed study protocol, all participating women completed the given questionnaires:

### Profile of Mood States (POMS)

It is a self-reporting questionnaire measuring six mood conditions: tension-anxiety, anger-hostility, fatigue-inertia, depression-dejection, vigour-activity and confusion-bewilderment [17, 18]. The woman is asked to indicate how intensely on a 5-point Likert-type scale ranging from 0 (*not at all*) to 4 (*extremely*) she has experienced a list of 58 feelings during the past 7 days. POMS is largely used in perinatal literature, showing good psychometric properties [19] also in Italian women population [20]. In the present study internal consistency of single subscales ranges from Cronbach’s alpha = 0.69 (tension-anxiety) to Cronbach’s alpha = 0.88 (vigour-activity).

### Socio-demographic and clinical data questionnaire

Socio-demographic data (age, education, marital status), obstetric and clinical data (parity, mode of conception, previous miscarriages or abortions, and childbirth (vaginal/caesarean section) were collected. In addition, all women were asked through a multiple-choice question which feeding practice they were using with their baby: breastfeeding, formula feeding or mixed feeding. The clinical group also filled out a cancer information leaflet (tumour site, treatment, age at time of diagnosis).

### Data analysis

Descriptive statistics, such as counts, percentages and means, were used to specifically analyse both the clinical and control group. Differences between two groups about socio-demographic characteristics and pregnancy data were analysed through Chi square test and independent sample T test. Chi square test was also used to examine the differences in feeding practices between clinical and control group. Due to the non-normal distribution of some sub-scales of the POMS, the relationship between emotional adjustment (the six dimensions of POMS) and three different feeding practices in clinical and control groups were assessed through the independent-samples Kruskal–Wallis test.

## Results

Inferential statistics in socio-demographic characteristics of clinical and control groups are illustrated in Table 1. In our series, the women from the two groups did not differ with regard to years of education, marital status, parity, previous miscarriage, pregnancy onset. However, women from the oncological series were older than controls. Notably, women in the clinical sample received their cancer diagnosis in their 30s and had to complete their oncological treatment before attempting a pregnancy. With reference to diagnosis details in the clinical sample, 82.4% (*N* = 28) of the women had received a breast cancer diagnosis, 5.9% (*N* = 2) gynaecological cancer, 5.9% (*N* = 2) hematologic cancer, 2.9% (*N* = 1) lung cancer and 2.9% (*N* = 1) PEComa.

### Aim 1

The chi-square test shows a statistically significant difference (*χ*^2^ = 19.73, df = 2, *p* = 0.001) in feeding practices between the two groups. Specifically, women in the clinical group breastfeed significantly less than women in the control group at three months age of the infant. Accordingly, women with an oncological history tend to use formula feeding more than mothers in the control group (Table 2).

### Aim 2

The Kruskal–Wallis test was conducted separately for clinical and control group. In the clinical sample a statistically significant relationship between feeding methods and mood state confusion emerged (*H* = 6.01; *p* = 0.04): breastfeeding mothers with a history of cancer feel significantly more confused (mean = 9.50; SD = 3.32) than oncological mothers who bottle-feed or use mixed feeding methods (mean = 5.23; SD = 4.00). In the control group, a significant association emerged between feeding methods and vigour (*H* = 6.00; *p* = 0.05); specifically, women who breastfed felt significantly more vigorous (mean = 19.37; SD = 5.19) than women who bottle-feed or use mixed methods (mean = 15.10; SD = 5.97) (Table 3).

## Discussion

Our work explores the breastfeeding behaviour in mothers with previous oncological history compared to non-cancer population and to analyse the association of this behaviour with mood states.

Our results clearly show that mothers with cancer history choose breastfeeding significantly less than non-oncological ones at three months of age of a newborn. We hypothesise that such significant difference in feeding behaviour may be due to the information generally provided to all young mothers referring to UNICEF ‘Breastfeeding Promotion and Support’ guidelines that recommend exclusive lactation until 6 months of the baby’s age and complementary food with continued breastfeeding up to 2 years of age [15]. Although useful for the majority of puerperal women, these guidelines may be perceived as non-specific, not feasible or even inappropriate by mothers with a cancer history. They tend to consider this general information not targeted and not applicable to their clinical history. In particular, it must be noted that women with a history of breast cancer who undergo surgery may encounter problems with breastfeeding [8, 14, 22] that leads them to single breastfeeding. Taking all these aspects into account, and considering that our study group was composed mainly of women with previous breast cancer, our data confirm that, within mothers with oncologic history, those who breastfeed reported higher levels of psychological distress and confusion compared to those who bottle-feed or use mixed feeding methods. It must be remarked that the questionnaire used to assess the women’s mood state (POMS) asks to indicate the intensity of symptoms without specifying the reason. Thus, although we did not know the reason why clinical women are more stressed or confused, we can suppose that their mood states are linked to the problem reported in breastfeeding also due to the lack of target information in line with a previous study [11]. Gorman [11] in fact showed that women with breast cancer face considerable challenges in breastfeeding, especially due to the fact that they relied mostly or entirely on one lactating breast. On the other side, in our series *puerperae* without a cancer diagnosis who choose to breastfeed show higher levels of vigour and activity, compared to those who prefer bottle-feeding or mixed feeding methods. A potential selection bias in our results on the psychological experience of feeding behaviour may be associated with the fact that the clinical group is composed of multiparae in higher proportion compared to the control group (although the difference is not statistically significant). This difference may also reflect the older age of women with previous cancer diagnosis as opposed to the control group. For this reason, we cannot exclude that for the women in the clinical group previous experience with normal two-breast milking may increase confusion about feeding after cancer diagnosis. As a matter of fact, previous milking experience may constitute an additional confusing factor because, as suggested by Philips and colleagues [21], most multiparous women tend to repeat the exclusive breastfeeding of their first child for their second child.

These data confirm our assumption that mothers with a cancer history should not be left alone in their feeding choice. As shown, the lack of consensus among healthcare workers and clear information for mothers about breastfeeding after cancer, a mother with oncologic history may feel alone in the choice trying to repeat a past experience, although aware that their health condition has changed. In addition and regardless of the parity, mothers with previous cancer history may be exposed to contradictory messages and perceptions circulating in the general public. On one hand, in fact, this particular group of mothers, as well as all puerperal women, are made subject to social pressure focusing on the advantages of breastfeeding for infant health and for the mother–baby relationship. In contrast, they might be aware and genuinely concerned about the adequacy of their milk for infant feeding and for their health. It is very likely that mothers with previous oncology disease and treatment believe that they are not able to produce an adequate quantity of milk to feed their infant or alternatively that their milk quality is not so good for infant health as that produced by a healthy mother. It is worth reiterating and emphasising that breast cancer deserves particular attention in the context of breastfeeding after an oncological disease. It is evident that breast cancer survivors face unique challenges when they attempt breastfeeding [14], from both a medical and psychological perspective. The oncology literature underlines that milk production is significantly reduced in the breast previously treated with breast-preserving surgery and lumpectomy if the nipple or areolar complex was involved [14, 22]. Similarly, even when milk production is still maintained from the irradiated breast, breastfeeding should be avoided as mastitis is more difficult to treat in the breast which has been irradiated. However, there is no reported evidence in the current literature showing compromised milk production from the contralateral breast [14]. Therefore, puerperal women previously treated for breast cancer who do not show any evidence of residual tumour should be reassured by clinicians about the adequacy of the one-breastfeeding option for the nutritional need of the newborn [10]. Nevertheless, even if one-breastfeeding is technically possible, these mothers who take on this choice should be psychologically supported with tailored and focused initiatives. Accordingly, psychological counselling remains a crucial point that could have a significant impact on the success of breastfeeding in women with a cancer history.

## Conclusions

Our study advocates the need for a specific *warm chain* of support for women with a cancer history in order to reduce their confusion around breast-feeding. In this respect, it is of utmost importance to help mothers to express their anxieties, fears, and concerns about how to feed their child. Health professionals should be prepared to discuss with the mother the feasibility of breastfeeding and the psychological consequences of this choice in terms of maternal stress and anxiety. We urge further studies and scientific discussion to overcome the current lack of robust clinical data and wide consensus among healthcare professionals. Future investigations should better explore the dimension of confusion of women with past cancer by mean of interviews or qualitative data, in addition to the questionnaire. This would facilitate developing too long-awaited guidelines in this subject to support and counsel women in their decision.

## Figures and Tables

**Table 1.** Maternal demographic characteristics and information about pregnancy.

			Clinical sample	Control sample	Diff.
			*N* = 34	*N* = 40	
*Demographic characteristics*					
Age (years)	*M* (SD)		38.61 (3.64)	32.11 (5.38)	*t* = 5.87; df = 72 *p* < 0.001
	range		31–46	22–47	
Marital status	% (*N*)	Married/ cohabiting	100 (34)	95 (38)	*χ*^2^ (1,74) =1.84; *p* = 0.175
		Separated/divorced	0	5 (2)	
Education (years)	*M* (SD)		16.6 (3.45)	16.27 (3.71)	*t* = 0.475; df = 72; *p* = 0.636;
*Information about pregnancy*					
Parity	% (*N*)	Primiparous	76.5 (26)	85 (34)	*χ*^2^ (1,74) = 0.87; *p* = 0.35
		Pluriparous	23.5 (8)	15 (6)	
Previous miscarriages	% (*N*)	Yes	11.8 (4)	22.5 (9)	*χ*^2^ (1,74) = 1.46; *p* = 0.227
		No	88.2 (30)	77.5 (31)	
Pregnancy onset	% (*N*)	Naturally	85.3 (29)	92.5 (37)	*χ*^2^ (1,74) = 0.99; *p* = 0.32
		IVF	14.7 (5)	7.5 (3)	
*Information about past cancer*					
Oncologic disease	% (*N*)	Gynaecologic	5.9 (2)		
		Breast	82.4 (28)		
		Hematologic	5.9 (2)		
		Lung	2.9 (1)		
		PEComa	2.1 (1)		
Treatment	% (*N*)	Chemotherapy	73.3 (22)		
		Radiotherapy	53.3 (16)		
		Surgery	61.3 (19)		
		Hormones	56.7 (19)		
Age at diagnosis	*M* (SD)		31.35 (4.38)		
	Range		24–40		

**Table 2.** Crosstab samples*feeding methods.

		Breastfeeding	Formula	Mixed method
Clinical sample	*N*	4	21	9
	%	11.8%	61.8%	26.5%
Control sample	*N*	20	6	14
	%	50.0%	15.0%	35.0%
Total	*N*	24	27	23
	%	32.4%	36.5%	31.1%

**Table 3.** Mean (SD) of POMS total score and subscales in clinical and control sample related to feeding method.

	Clinical sample		Control sample	
	Breastfeeding	Formula/Mixed method	Breastfeeding	Formula/Mixed method
POMS total	49 (20.26)	42.9 (14.59)	45.89 (12.21)	52.3 (24.53)
POMS anxiety-tension	6.5 (4.50)	6.33 (64.22)	6.57 (4.0)	8.85 (5.66)
POMS anger-hostility	3 (2.44)	3.96 (4.23)	3.26 (3.96)	5.95 (8.0)
POMS fatigue-inertia	8 (4.08)	5.70 (4.27)	5.52 (2.71)	7.50 (4.24)
POMS depression-dejection	4.25 (4.78)	2.66 (4.12)	3.36 (3.81)	3.33 (3.55)
POMS vVigour-activity	17.75 (7.88)	19.16 (6.22)	19.36 (5.18)	15.10 (5.97)
POMS confusion-bewilderment	9.5 (3.31)	5.23 (4.00)	7.78 (3.25)	9.4 (5.63)

## References

[1] Kaiser AM (1994). Warm chain for breastfeeding. Lancet.

[2] Kramer M, Kakuma R (2012). Optimal duration of exclusive breastfeeding (review). Cochrane Database Syst Rev.

[3] Collaborative Group on Hormonal Factors in Breast Cancer (2002). Breast cancer and breastfeeding: collaborative reanalysis of individual data from 47 epidemiological studies in 30 countries, including 50 302 women with breast cancer and 96 973 women without the disease. Lancet.

[4] Ip S, Chung M, Raman G (2007). Breastfeeding and maternal and infant health outcomes in developed countries. Evd Rep/Tech Assess.

[5] Semenic S, Childerhose JE, Lauziere J (2012). Barriers, facilitators, and recommendations related to implementing the Baby‐Friendly Initiative (BFI): an integrative review. J Hum Lact.

[6] Zakarija-Grković I, Cattaneo A, Bettinelli ME (2020). Are our babies off to a healthy start? The state of implementation of the Global strategy for infant and young child feeding in Europe. Int Breastfeed J.

[7] AIOM (2018). Breast neoplasm guidelines. https://www.aiom.it/en/aiom-scientific-publications/.

[8] Azim HA, Bellettini G, Gelber S (2009). Breast-feeding after breast cancer: if you wish, madam. Breast Cancer Res Treat.

[9] McCullough L, Ng A, Najita J (2010). Breastfeeding in survivors of Hodgkin lymphoma treated with chest radiotherapy. Cancer.

[10] Azulay Chertok IR, Wolf JH, Beigelman S (2020). Infant feeding among women with a history of breast cancer. J Cancer Surviv.

[11] Gorman JR, Usita PM, Madlensky L (2009). A qualitative investigation of breast cancer survivors’ experiences with breastfeeding. J Cancer Surviv.

[12] Lee E (2007). Health, morality, and infant feeding: British mothers’ experiences of formula milk use in the early weeks. Sociol. Health Illn.

[13] Knaak SJ (2010). Contextualizing risk, constructing choice: breastfeeding and good mothering in risk society. Health RiskSoc.

[14] Azim HA, Peccatori FA, de Azambuja E (2011). Motherhood after breast cancer: searching for la dolce vita. Breast Cancer.

[15] UNICEF (2018). Breastfeeding. A Mother’s Gift, for Every Child.

[16] Mascheroni E, Faccio F, Bonassi L (2019). Exploring differences in psychological aspects during pregnancy between cancer survivors and women without a history of cancer. Support Care Cancer.

[17] McNair DM, Lorr M, Droppleman LF (1971). Manual for the profile of mood states (POMS).

[18] Farnè M, Sebellico A, Gnugnoli D (1991). POMS: Profile of Mood States.

[19] Groer MW, Morgan K (2007). Immune, health and endocrine characteristics of depressed postpartum mothers. Psychoneuroendocrinology.

[20] Ionio C, Colombo C, Brazzoduro V (2016). Mothers and fathers in NICU: the impact of preterm birth on parental distress. Eur J Psychol.

[21] Phillips G, Brett K, Mendola P (2011). Previous breastfeeding practices and duration of exclusive breastfeeding in the United States. Matern Child Health J.

[22] Moran MS, Colasanto JM, Haffty BG (2005). Effects of breast-conserving therapy on lactation after pregnancy. J Cancer.

